# Monitoring and investigation of tachycardia in patients receiving clozapine therapy; a quality improvement project

**DOI:** 10.1192/bjo.2021.542

**Published:** 2021-06-18

**Authors:** Michael Kurkar, Joshua Blair, Poppy Graham

**Affiliations:** Royal Oldham Hospital, Oldham, Pennine Care NHS Foundation Trust

## Abstract

**Aims:**

Clozapine is an antipsychotic agent with a number of significant physical health risks which necessitate monitoring, including cardiac complications such as myocarditis and cardiomyopathy. Reliable detection of cardiac complications requires active vigilance, with consideration for investigations if serious cardiac side effects are suspected (including electrocardiograms, echocardiograms and blood tests). There was dissatisfaction in the outpatient department about delays to taking action on abnormal physical observations, such as tachycardia. This raised safety concerns about how these delays would limit our ability to investigate and diagnose cardiac complications in a timely manner. We set a project aim to reduce the rate of retrospective action on abnormal physical observations, by half in the 4-month project timespan.

**Method:**

All correspondence sent to the outpatient department from the local clozapine clinic was monitored and assessed for the need for further action or investigation, and the proportion of retrospective action needed was recorded. This was then monitored during implementation of project interventions, to detect any change in performance.

**Result:**

Baseline monitoring showed retrospective action had to be taken on 41.2% of patients attending the clinic with abnormal physical observations, with significant delays up to 51 days later. Our initial intervention was the design of a clinical protocol to guide and signpost clinical staff at the time of the patient's attendance. Unfortunately, due to wider organisational barriers, this was not able to be implemented during the timescale of this project; however increased staff awareness during the protocol implementation process led to a reduction of retrospective action to 26.7%. A follow-up intervention to increase staff awareness and education was carried out, with development of a poster for the clinical room. This approach maintained the improvement, with a further slight reduction to 26.3%, representing a decrease of 37.5% from the baseline rate. A total of 106 patient letters were assessed during the project.

**Conclusion:**

We believe that developing a clinical policy to use at the time of the patient's clinic attendance still remains the optimal intervention; a view backed up by this project's identified drivers for change. However, wider organisational barriers prevented the implementation of this policy, and overcoming these barriers are outside the scope and timescales of this project. This project demonstrated maintained, but sub-target, success with measures that increase staff education and awareness. However, it remains to be seen if this improvement will persist, and this would be a potential target for further QIP or monitoring.

